# Supplementary figures and images for: TNIP1 and Autophagy Receptors regulate STING Signaling
Source: bioRxiv. 2025 Apr 23:2025.04.21.649822. Preprint. [Version 1] doi: 10.1101/2025.04.21.649822 (PMC12190477; doi:10.1101/2025.04.21.649822)

# Figure S1

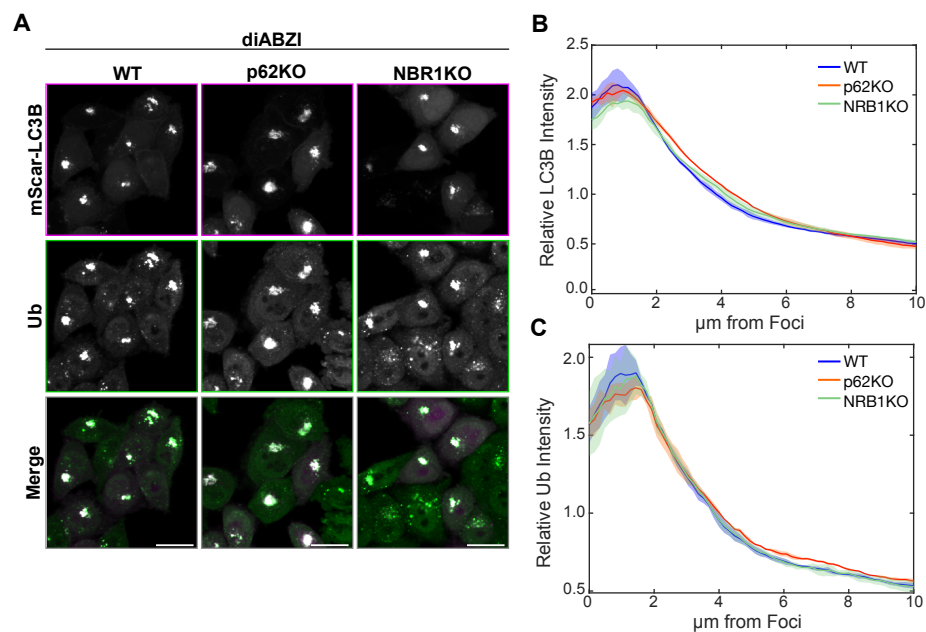

# Figure S2

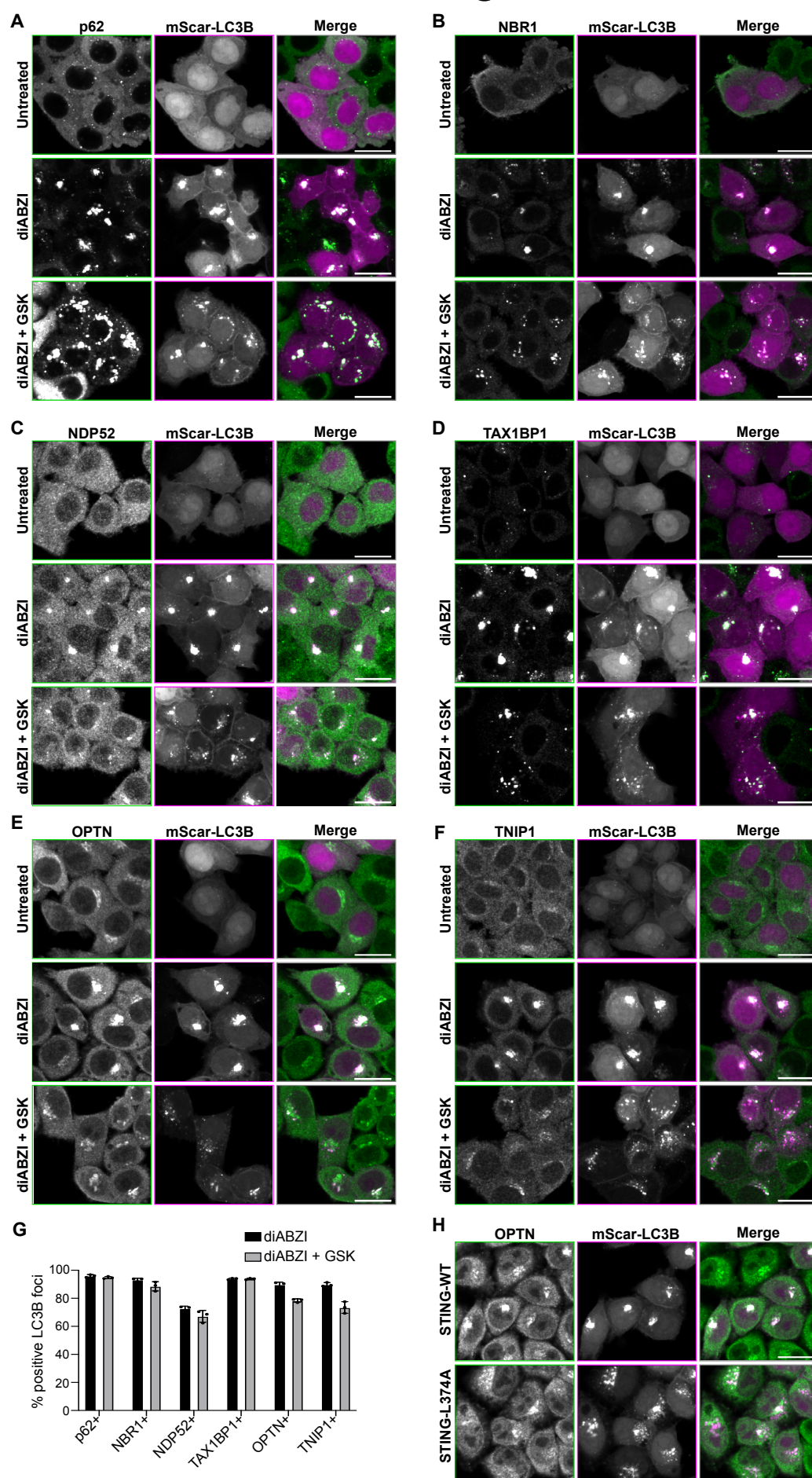

## Figure S3

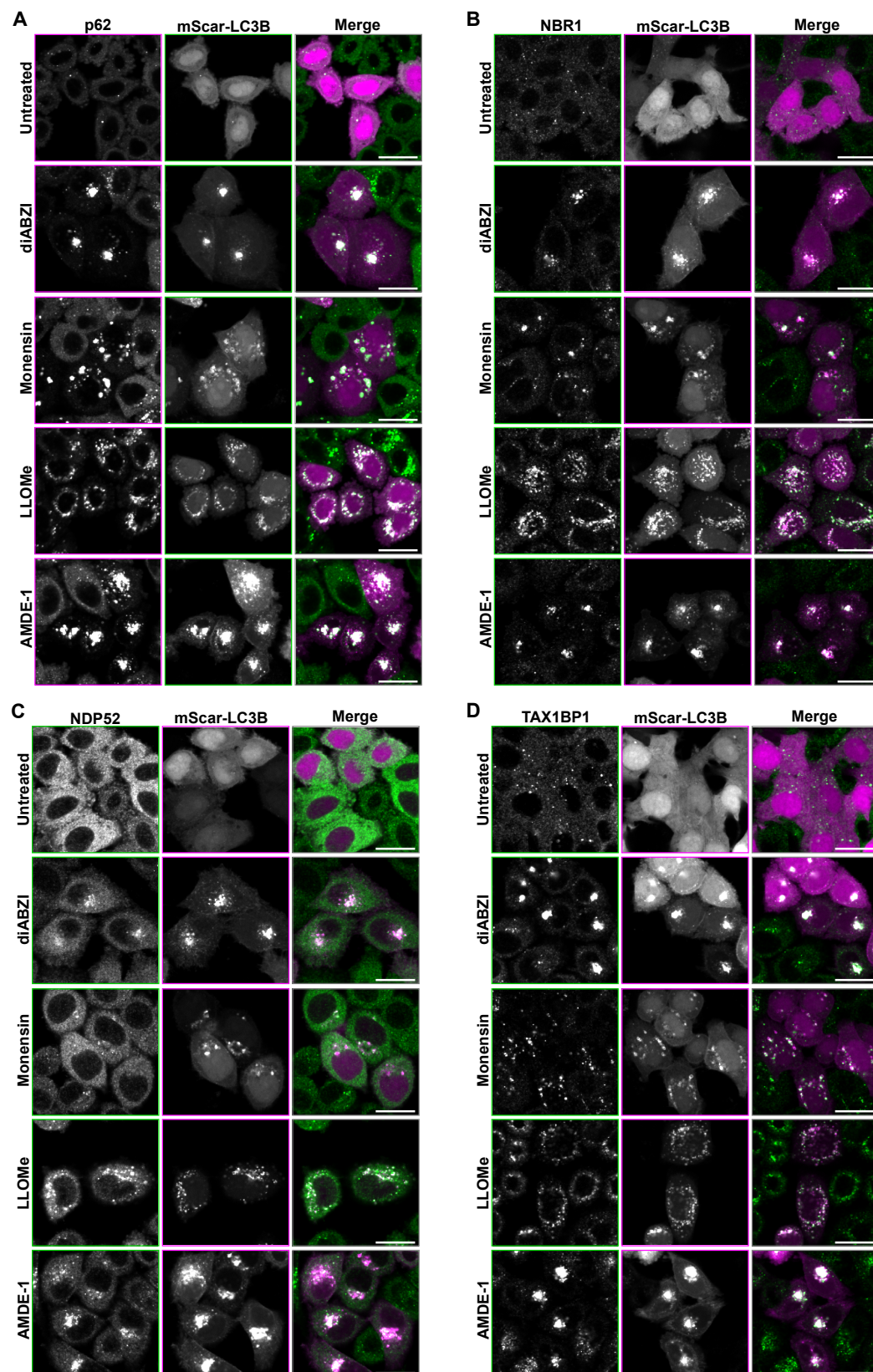

Figure S4

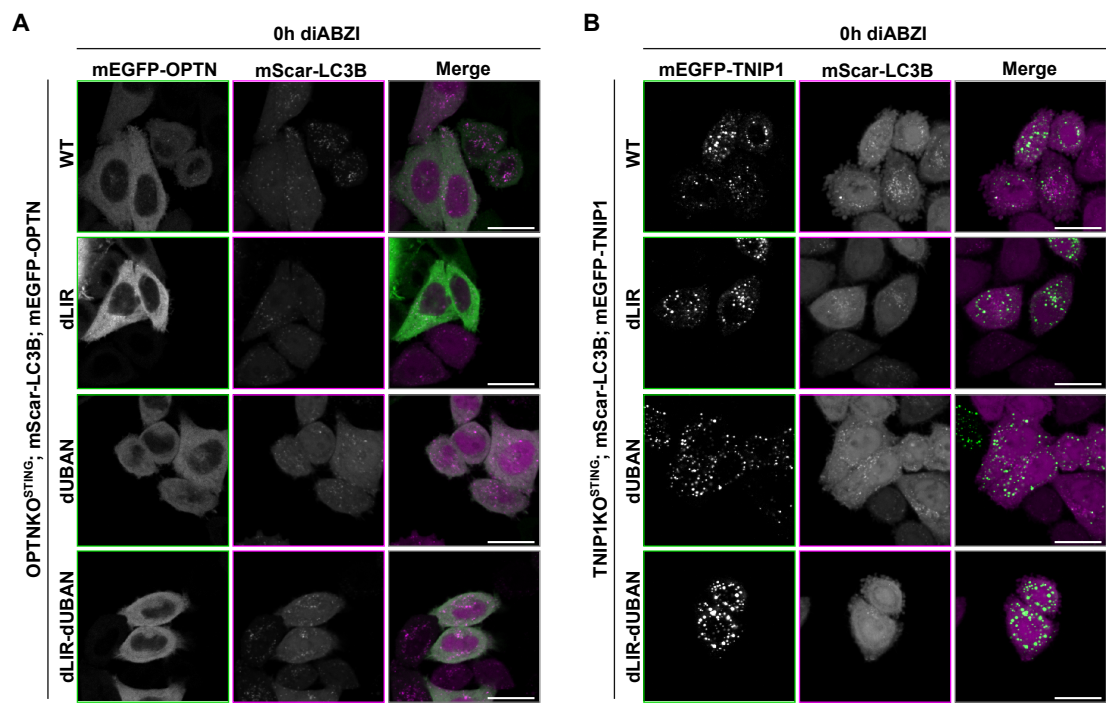

## Figure S5

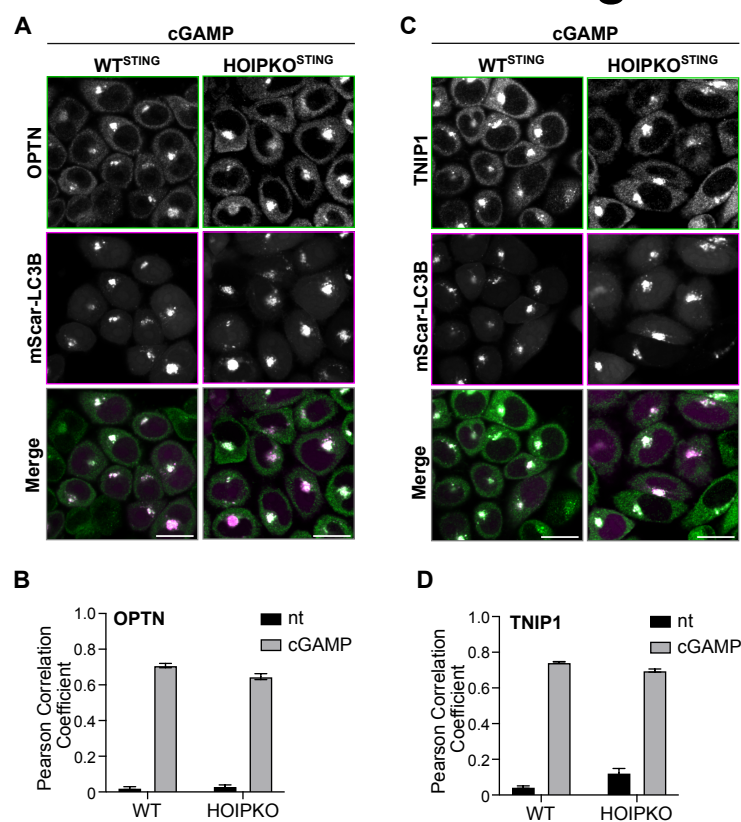

Supplement: 1 — Figure S1. A) Representative spinning disk confocal images of WT, p62KO, or NBR1KO HeLaSTING cells expressing mScarlet-LC3B (magenta) treated for 6 hr with diABZI then fixed and immunostained for ubiquitin (green). Scale bars indicate 20 μm. B-C) Normalized intensity of LC3B (B) or signal within each cell plotted against distance to centroid of LC3B signal from cells displayed in panel A. Over 1000 cells were imaged for each condition. Figure S2. A-F) Representative spinning disk confocal images of WT HeLaSTING cells expressing mScarlet-LC3B (magenta) treated for 6 hr with diABZI or diABZI and TBK1 kinase inhibitor GSK, then fixed and immunostained for indicated proteins (green). Scale bars indicate 20 μm. G) Quantification of images in panels A-E depicting the fraction of LC3B foci positive for indicated protein. Over 1000 cells were imaged for each condition. H) Representative spinning disk confocal images of WT HeLaSTING or HeLa with STINGL374A expressing mScarletI-LC3B (magenta) treated for 6 hr with diABZI, then fixed and immunostained for OPTN (green). Scale bars indicate 20 μm. Figure S3. A-D) Representative images of WT HeLaSTING cells expressing mScarletI-LC3B (magenta) and immunostained for indicated proteins (green) after no treatment, diABZI (6 hr), Monensin (1 hr), LLOMe (1 hr), or AMDE-1 (6 hr). Scale bar indicates 20 μm. Figure S4. A-B) Representative spinning disk confocal images of OPTNKO (A) or TNIP1KO (B) HeLaSTING cells stably expressing mScarletI-LC3B and either mEGFP-OPTN- (A) or TNIP1- (B) WT, dLIR, dUBAN, or dLIR-dUBAN at the 0-hour and 6-hour time points represented in Figure 5, C–D following treatment with 1uM diABZI. Scale bar = 20 μm. Figure S5. A) Representative spinning disk confocal images of WT and HOIPKO HeLaSTING cells stably expressing mScarI-LC3B (magenta) cells treated with 120 μg/mL of cGAMP for 8 hours prior to PFA-fixation and immunostaining for OPTN (magenta). Scale bar = 20 μm. B) Pearson correlation coefficient of mScarI-LC [file NIHPP2025.04.21.649822V1-supplement-1.pdf]
